# The Hands

**Published:** 1869-09

**Authors:** M. McCarty


### THE HANDS.

EDITORS DENTAL REGISTER:—I noticed a communication in the June number of the REGISTER, headed "The Hands," by C. H. EVANS, in which he speaks of the difficulty of keeping the hands in a proper condition to operate when we have to handle blackened flasks just from the vulcanizer. He goes on and gives the best remedy for its removal. I admit that it does it admirably, but there is an old adage that says "an ounce of prevention is worth a pound of cure." Here it is, how to prevent blackening of the hands.

Prepare a strong solution of lime of sufficient quantity to cover the flasks well in a vessel suitable for the purpose. Take the flasks from the vulcanizer with a pair of tongs, put them in the solution, allow them to remain say fifteen minutes, stirring a few times. Remove them with the tongs and place them in clean water when they may be washed off with a brush. After all the plaster has been washed off the flasks they should be replaced in the solution, and allowed to remain half an hour or more. As this will prevent them from rusting, and their consequent early destruction. The black coating on the flasks is a compound formed of the sulphuric acid of the plaster, with the iron of the flasks, together with some of the volatilized rubber. The sulphuric acid is set free by the high degree of heat used in vulcanizing. Also, it is likely the sulphur in the rubber plays some part. When the flasks are thrown in the lime solution, the sulphuric acid leaves the iron and unites with the lime, while the iron is precipitated as an inert black powder. Other strong alkalies might be used that have an affinity for sulphuric acid. The reason for using the tongs to handle the flasks is apparent, as the lime solution would injure the hands worse than the black and the remedies for its removal. M. McCarty.

